# Plant roots: new challenges in a changing world

**DOI:** 10.1093/jxb/erw027

**Published:** 2016-02-13

**Authors:** Adam H. Price

**Affiliations:** University of Aberdeen

As research on roots intensifies due to the growing realization that a better understanding of their function and genetics offers important strategies for tackling the global challenge set by increased population, climate change, and food security, it is important to present new developments in the area. This special issue features articles offered by contributors to a session on that subject at the Society of Experimental Biology Annual Meeting in Prague in 2015 which was sponsored by European Union project called EURoot (www.EURoot.eu).

The review by Berg *et al.* (2016) reviews current knowledge on the role of microorganisms in the phyllosphere (above ground), rhizosphere (soil close to roots), and endosphere (inside the plant) in plant growth, development, and function and highlights the need to consider metaorganisms which are co-evolved assemblages of plant and microbes working together. The work argues for a change in consideration of what a plant is and highlights the diversity of the microorganisms involved, the transmission between plants, the importance of endophytes, and several unexpected functions and metabolic outcomes of the biological interactions of the metaorganism.

The first survey of the drought response of roots at the transcriptome level was the subject of a review by Janiak *et al.* (2016). Since it is the root that initially experiences and signals drought to the plant, this is a valuable collation of literature which highlights the important role of several classes of transcription factors, the downstream targets, and the interplay with hormones and other signalling molecules. A related review by Shabala *et al.* (2016) focuses on the role of membrane transporters in the sensing and response to soil-based plant stresses first experienced by the root—those of drought, salinity, and flooding, emphasizing specific genes with promise for conferring tolerance. It highlights the importance of maintaining membrane potential and ATP production during stress and the role of reactive oxygen species and cytosolic Ca^2+^ and K^+^ in signalling responses while also detailing the role of ion transporters in the response to these stresses.

Current root research has placed a lot of effort into improving phenotyping that is either more accurate, more high throughput or more relevant. Addressing these issues, Wasson *et al.* (2016) provide details of a new portable fluorescence spectroscopy imaging system that can be used in the phenotyping of roots that improves accuracy and speed and reduces costs. Importantly, this is applied to a crop (wheat) grown in the field which is the most relevant environment in which to phenotype roots, but where root research is rarely conducted due to the high variation and high labour requirements.

Another major thrust of root research is the development and improvement of models that can aid in the characterization of plant form and function and help produce better target traits for specific crop improvements. Kalogiros *et al.* (2016) describes a density-based model of root growth that can back-calculate root parameters based on images taken at one time point. The authors show that the model predicts root traits with high accuracy in a diverse panel of *Brasica rapa*, and suggest that the model will be useful in identifying genotypes with efficient root systems in phenotyping screens even if they have incomplete image data. Concentrating more on root function, a model of nutrient uptake by root hairs is presented by Daly *et al.* (2016). Using data from X-ray Computed Tomography the authors model the distance into the bulk soil that is required for root hairs to be ineffective (and hence for root hair distribution to be irrelevant) and find that, for relatively immobile nutrients like phosphorus, it is less for saturated soil (1.1mm) than partially saturated soil (1.4mm).

Two further papers consider the role of root hairs. Bengough *et al.* (2016) used time-lapse photography of a root hairless mutant of maize and its wild type to calculate the anchorage force afforded by root hairs. It provides an insight into the role of root hairs in anchoring roots and shows the considerable advantage they provide in allowing penetration of weak and medium penetration-resistance soils, but not hard soils. Using a root hairless barley mutant and its wild type, Kwasniewski *et al.* (2016) investigated the role of the root hair in the signalling of drought stress. They used a transcriptomics approach. The results suggest that, while the mutant showed most of the typical responses to drought, they appear to have less of the responses associated with root to shoot stress signalling, suggesting a role for root hairs in drought perception and/or signalling.

The pattern of gene expression in different tissues of the root was investigated by Optiz *et al*. (2016). These authors split the maize root into four different sections, being the tip, the elongation zone, and then the cortex and stele of the mature zone. The transcriptomic responses to PEG-induced drought were different in these sections, highlighting their different roles and reactions for sensing, signalling, and responding to drought. Another article details the effect of drought on gene regulation, but this time on the methylation status of genes. Chwialkowska *et al.* (2016) assessed the methylome of the leaves and roots of barley seedlings exposed to drought and after recovery using Methylation Sensitive Amplification Polymorphism Sequencing. The results showed evidence of methylation and demethylation in response to drought which was balanced in leaves but was dominated by methylation in roots where recovery to the pre-drought state appeared slower. One further article focuses on gene expression. Tai *et al.* (2016) used transcriptomics and anatomical measurement to differentiate three different types of maize root, the embryonic primary and seminal roots, and the post-embryonic crown roots. Although gene expression differences were relatively small (in the order of 1% of genes differentially expressed between root types), global expression profiling suggests that those genes that were different between tissues reveal differences in root function.

Researchers from the same laboratory as above (Zhang *et al.*, 2016) have investigated the role of two genes known to be involved in lateral and seminal root production in maize, *RUM1* (*ROOTLESS WITH UNDETECTABLE MERISTEM 1*) and its homeologue *RUL1* (*RUM1-like 1*), both of which are regulators of auxin signalling. While both genes have similar molecular properties, the expression of *RUL1* is much lower than that of *RUM1* while the authors reveal a RUM1-associated protein 1 (RAP1) that specifically interacts with RUM1 but not with RUL1 suggesting that RUM1 and RUL1 are at least partially associated with different molecular networks.

The gene *RUM1* also featured in one of two papers that examine the detection and/or utility of quantitative trait loci (QTLs) detected for root system architecture traits. In Salvi *et al.* (2016), three QTLs for the number of roots were detected in maize introgression lines, with two of these being above excellent candidate genes, *RUM1* and *RTCS* (*rootless concerning crown and seminal roots*). In durum wheat, Maccaferri *et al.* (2016) used two linkage mapping populations and one genome-wide association panel (with about 90K markers) to identify root trait QTLs, revealing several genomic regions with clusters of QTLs for root length and number or root angle detected by both mapping approaches. In both of these papers the authors discuss the implications of these QTLs for breeding.

Genome-wide association mapping was used in rice for the final two papers of this special issue. A total of 196 accessions were assessed for root and shoot growth under low phosphorus by Mori *et al.* (2016) revealing major differences in root efficiency of phosphorus uptake (amount of P taken up per unit of root surface area). Detailed physiology revealed several-fold differences in root efficiency between cultivars while mapping revealed, among others, a QTL on chromosome 11 with major potential for crop improvement. Finally, Dimkpa *et al.* (2016) screened 330 rice accessions for resistance to the root-knot nematode *Meloidogyne graminicola* revealing 11 QTLs and some notable candidate genes. They also discovered two rice landraces with complete resistance to the nematode which was confirmed in separate, large-pot experiments in two independent laboratories, providing excellent breeding strategies for improving resistance to this soil-borne pest that severely impacts root growth and function.

Together these reviews and original research papers provide a glimpse into some of the issues and methodologies that are occupying root scientists at the current time.
